# Influence of Cell Type in In Vitro Induced Reprogramming in Cattle

**DOI:** 10.3390/life12081139

**Published:** 2022-07-28

**Authors:** Kaiana Recchia, Laís Vicari de Figueiredo Pessôa, Naira Caroline Godoy Pieri, Pedro Ratto Lisboa Pires, Fabiana Fernandes Bressan

**Affiliations:** 1 Department of Surgery, Faculty of Veterinary Medicine and Animal Science, University of São Paulo, Av. Prof. Dr. Orlando Marques de Paiva, 87, São Paulo 05508-270, SP, Brazil; 2 Department of Veterinary Medicine, Faculty of Animal Science and Food Engineering, University of São Paulo, Av. Duque de Caxias Norte, 225, Pirassununga 13635-900, SP, Brazil; laisvpessoa@usp.br (L.V.d.F.P.); nairagodoy@usp.br (N.C.G.P.); pedroratto@usp.br (P.R.L.P.)

**Keywords:** bovine, cellular reprogramming, multipotent, pluripotent, iPSCs

## Abstract

Induced pluripotent stem cells (iPSCs) have been considered an essential tool in stem cell research due to their potential to develop new therapies and technologies and answer essential questions about mammalian early development. An important step in generating iPSCs is selecting their precursor cell type, influencing the reprogramming efficiency and maintenance in culture. In this study, we aim to characterize bovine mesenchymal cells from adipose tissue (bAdMSCs) and fetal fibroblasts (bFFs) and to compare the reprogramming efficiency of these cells when induced to pluripotency. The cells were characterized by immunostaining (CD90, SSEA1, SSEA3, and SSEA4), induced differentiation in vitro, proliferation rates, and were subjected to cell reprogramming using the murine OSKM transcription factors. The bFFs presented morphological changes resembling pluripotent cells after reprogramming and culture with different supplementation, and putative iPSCs were characterized by immunostaining (OCT4, SOX2, NANOG, and AP). In the present study, we demonstrated that cell line origin and cellular proliferation rate are determining factors for reprogramming cells into pluripotency. The generation of biPSCs is a valuable tool to improve both translational medicine and animal production and to study the different supplements required to maintain the pluripotency of bovine cells in vitro.

## 1. Introduction

Stem cells have two main characteristics, the capacity for self-renewal and the potential to differentiate into other cell types, and are widely studied for their use in veterinary and human regenerative medicine [1,2]. The potential for cell differentiation varies according to the classification of the stem cells, where the totipotent cell (zygote) can be differentiated into embryonic germ layers and extra-embryonic tissues [3], and the pluripotent cells can differentiate into tissues derived from the three germinative layer origins (endoderm, mesoderm, and ectoderm) and in progenitor germ cells (PGCs) [4,5], whereas multipotent cells present limited plasticity and usually can only be differentiated in cells derived from the same germinative layer tissue; for example, mesenchymal cells derived from the mesoderm are capable of differentiating into osteocytes, adipocytes, or chondrocytes [6,7]. The self-renew and multipotent characteristics could facilitate the reprogramming process once those cells have a few endogenous pluripotency-inducing factors in abundance [8].

Induced pluripotent stem cells (iPSCs) were first reported from murine, followed by human fibroblasts, transduced with a retroviral vector containing the transcription factors OCT4, SOX2, KLF4, and c-MYC (OSKM) [9,10]. The reprogramming of somatic cells has become a valuable source of pluripotent cells and has been established for several species of domestic and wild animals, including large animals such as swine [11,12,13,14], equine [15,16], buffalo [17], and bovine [4,18,19,20].

The generation of iPSCs in the bovine model (biPSCs) can greatly benefit veterinary and human regenerative medicine studies, serving as a biomedical model or bioreactors [2,21]. It can also be applied to elucidate further the mechanisms involved in embryonic development [18] and reproductive technologies [2,22,23,24], aiming at the acquisition of genetically superior animals [5]. The biPSCs already reported were generated from the adult, fetal, or embryonic fibroblasts [25,26,27], amniotic cells [20], mammary cells [28], and Sertoli cells [19]. The biPSCs were generated through viral vectors [18,19,25,26,28], PiggyBac transposon [20,27], and electroporation [29] using OSKM from murine [18,28], human [19], or bovine transcription factors [27], and occasionally with the addition of NANOG and LIN28 [26,30].

To characterize the biPSCs, the detection of usual pluripotent markers, including mainly OCT4, SOX2, and NANOG, and differentiation into embryoid bodies or teratoma assays are usually performed, and the standard gold test is the chimera production [20,31]. The biPSCs previously reported were generated in a divergent condition of culture in vitro, as reported for the supplementation with LIF (leukemia inhibitory factor) and BMP4 (bone morphogenetic protein 4) [25,29], or LIF and/or bFGF (basic fibroblast grow factor) [32]. Moreover, the precursor cells of iPSCs may influence the reprogramming methodology and the supplementation to be used; in humans, the comparison of different cell types shows that some, such as urine-derived cells (UDCs), can be more easily reprogrammed into pluripotent cells [32]. In humans, both the integration-free and integration-based reprogramming of keratinocytes, peripheral blood mononuclear cells, or UDCs has been reported due to their facilitated collection [33]; however, the most commonly used cells for pluripotency reprogramming in different animal species are still the mesenchymal stem cells, in addition to adult or fetal fibroblasts [34], which are traditional components of most cell biobanks. Thus, the selection of precursor cells is the first and crucial step for the acquisition of iPSCs aiming at efficient reprogramming.

Herein, two cell types, bovine fetal fibroblasts (bFF) and bovine adipose tissue mesenchymal stem cells (bAdMSC), were characterized and then reprogrammed using OSKM. The efficiency of reprogramming in different culture conditions (LIF or bFGF) was morphologically analyzed. Albeit further molecular analysis and an increased number of animals studied are needed to better support our findings, the results presented herein may contribute to the selection of cell origin, the establishment of a reprogramming protocol, and adequate culture conditions for biPSC generation and maintenance. The generated cells can be further used for the in vivo and in vitro study of diseases and genetic improvement, for example, for the in vitro generation of gametes and embryos.

## 2. Materials and Methods

All procedures were approved by the Ethics Committee on the Use of Animals of FZEA/USP (n° 3526250717). Unless otherwise stated, cells were cultured at 38.5 °C, 5% CO_2_, and maximal humidity (from here on, standard culture conditions), analyses were performed in biological triplicates, and photo-documentation was performed with a Nikon Eclipse TS100 microscope equipped with a Nikon DS-Ri1 camera and the NIS-Elements F (v 2.1) software.

### 2.1. Cell Culture

Briefly, bovine fetal fibroblasts (bFF) were isolated from a 50-day gestation fetus [18], and adipose tissue-derived mesenchymal cells (bAdMSC) were derived from 2 cm^3^ of adipose tissue [6] from an adult animal. The cells were maintained in T75 cm flasks using Iscove’s Modified Dulbecco’s Medium (IMDM, Life Technologies, Frederick, MD, USA) supplemented with 10% fetal bovine serum (FBS, Life Technologies) and 1% of penicillin and streptomycin (Life Technologies) at standard culture conditions. Cells were dissociated (TrypLE Express, Life Technologies) before reaching 80–90% confluency and cryopreserved at each passage.

### 2.2. Mesodermal In Vitro Differentiation

bFFs and AdMSCs were submitted to induced differentiation using the StemPro commercial kit (Cat#A10071-01, Life Technologies), as previously described [15] and according to the manufacturer’s instructions. For the chondrogenic cell differentiation, cells were plated in 5 μL IMDM droplets at a concentration of 1.6 × 10^7^ cells/mL and cultured under standard conditions for 2 h, when the differentiation medium was added and prepared according to the manufacturer’s instructions and refreshed every four days for two weeks. After the differentiation process, cells were fixed and stained using Alcian blue dye (Sigma-Aldrich, Taufkirchen, Germany) for chondrogenesis differentiation detection and were photo-documented.

### 2.3. Doubling Time Analysis

As previously described [14], for doubling time analysis, bFFS and AdMSCs were plated in 6-well plates at the initial density of 3 × 10^4^ cells/well and incubated for two days. Every 48 h, the cells were dissociated, counted using a Neubauer chamber, and reseeded at the initial density (3 × 10^4^ cells/well) [15]. This process was repeated nine times. The mean of cell doubling time (*DT*) was calculated in hours using the equation [35]:

$$DT=\frac{\left(T-T0\right)\cdot log2}{\left(logN-logN0\right)}$$

(*T* − *T*0): number of hours cells were cultured between passages, 

*N*0: initial cell density, 

*N*: number of cells harvested at each passage.

### 2.4. Immunocytochemistry for CD90, SSEA1, 3 and 4

bFFs and AdMSCs (8 × 10^3^ cells/well previously plated in 24-well plates and cultured until 70% confluency) were fixed with 4% paraformaldehyde. The cells were analyzed for typical mesenchymal marker CD90 (SC6071, Santa Cruz, CA, USA—1:200, secondary antibody A11078, Abcam, Cambridge, MA, USA—1:500), and for pluripotency markers: SSEA1 (MAB4301, Abcam—1:100), SSEA3 (FCMAB141A4, conjugated Abcam—1:50), and SSEA4 (MO813-70FCMAB116, conjugated Abcam—1:50). Briefly, the cells were washed with buffer (0.1% Tween + 1% Bovine Serum Albumin (BSA)). Subsequently, blocking with 10% BSA was performed, the cells were washed with buffer, and each well of the plate was incubated overnight with the specific primary antibody. After the incubation period, the cells were washed with phosphate buffer saline (PBS), incubated for 1 h with a secondary antibody (A11078, Abcam—1:500), washed with buffer, and incubated for 5 min with Hoechst (33342, Sigma-Aldrich, 1:100). Subsequently, washing with PBS was performed, and the cells were photo-documented as previously described.

### 2.5. Lentiviral Production and Cellular Reprogramming

The pluripotency induction of bFFs and AdMSCs was performed as previously reported [15,18]. The polycistronic lentiviral vector containing the murine reprogramming factors OCT4, SOX2, KLF4, and c-MYC was used (mOSKM-STEMCCA, Millipore, Burlington, MA, USA). To produce lentiviral particles, the lipofection protocol (Lipofectamine 3000, Life Technologies) was performed using auxiliary vectors TAT, REV, hgpm2, VSVG, and mOSKM in 293FT cells (Life Technologies). The bFFs and bAdMSCs were transduced overnight using polybrene 8 μg/mL (hexadimethrine bromide, Sigma-Aldrich) in 35 mm diameter dishes.

After six days, the cells were harvested using Tryple Express (Life Technologies) and 2 × 10^4^ cells/well were plated onto feeder layers of mitomycin C-treated mouse embryonic fibroblasts (MEFs) in iPSCs media composed of KnockOut Dulbecco’s modified Eagle’s medium (DMEM/F-12, Life Technologies), supplemented with 20% KnockOut Serum Replacement (KSR, Life Technologies), 1% L-glutamine (Life Technologies), 1% nonessential amino acids (Life Technologies), 1% antibiotics (pen/strep, Life Technologies), and 3.85 μM β-mercaptoethanol (Life Technologies). Media culture was supplemented with 10 μg/mL human bFGF (PeproTech, East Windsor, NJ, USA) or 1000 U/mL LIF (Merck, Temecula, CA, USA).

Cells were kept in iPSCs media for approximately 20 days or until colonies were formed, and the media was refreshed every two days. The efficiency of iPSC colony generation was measured by counting the number of colonies formed in relation to the number of cells initially plated to the transduction. After a first manual splitting of iPSC colonies, clonal lineages were enzymatically dissociated, replated, and cryopreserved. Reprogrammed cells were kept in culture for at least 21 passages at 38.5 °C and 5% CO_2_.

### 2.6. Characterization of biPSCs-Immunophenotyping

For alkaline phosphatase (AP) detection, the commercial Alkaline Phosphatase Detection Kit (86R-1KT, Sigma-Aldrich) was used. Briefly, colonies were fixed and analyzed according to the manufacturer’s instructions. Colonies stained in pink were considered positive. AP positive clonal lineages were further analyzed for the detection of NANOG, OCT4, and SOX2.

biPSCs colonies were washed with PBS, permeabilized with 0.1% Triton X for 20 min, washed with PBS, and blocked with 1% BSA/0.05% Tween for 1 h and labeled for 1 h with primary antibodies: NANOG (Ab21624, Abcam—1:100 dilution), OCT4 (Sc8628, Santa Cruz—1:100 dilution), and SOX2 (Ab97954, Abcam—1:250). Cells were then washed with 0.05% Tween, incubated for 1 h with a secondary antibody (anti-rabbit Alexa Fluor 488-1:500 dilution), washed as described above, and labeled with Hoechst 33342 (Sigma-Aldrich—1:1000 dilution) for 5 min. After a final wash with PBS, the cells were photo-documented.

## 3. Results

### 3.1. bFFs and bAdMSCs Analysis

**Figure 1:**
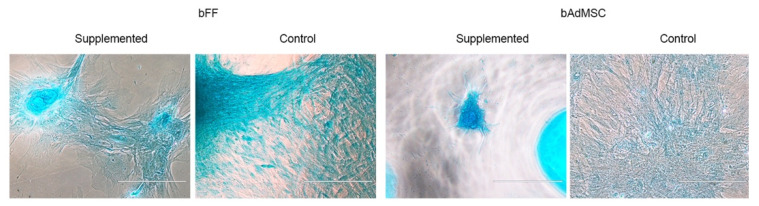
bFFs and bAdMSCs induced by chondrogenesis differentiation and control cells (Scale bar: 200 µm).

To evaluate the plasticity of bFFs and bAdMSCs, the cells were induced to chondrogenic lineage. The analysis of cell differentiation was performed based on the morphological comparison between the supplemented cells and the control cells (Figure 1). The cells submitted to chondrogenesis formed a cell mass stained by Alcian Blue, diverging from the control cells, which maintained their typical spindle-shaped morphology.

**Figure 2:**
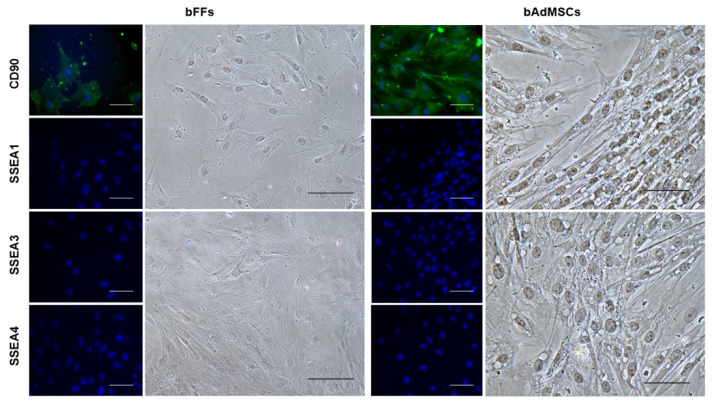
Positive detection of the CD90 marker and negative for SSEA-1, SSEA-3, and SSEA-4 in bFFs and bAdMSCs. Nuclei labeled with Hoechst. Scale bar 200 µm.

Immunocytochemistry was performed to detect pluripotent and multipotent markers in the bFFs and bAdMSCs. The CD90 marker (mesenchymal marker) was detected in both, but not the SSEA-1, SSEA-3, and SSEA-4 (putative markers of pluripotency; Figure 2).

### 3.2. Doubling Time Analysis

**Figure 3:**
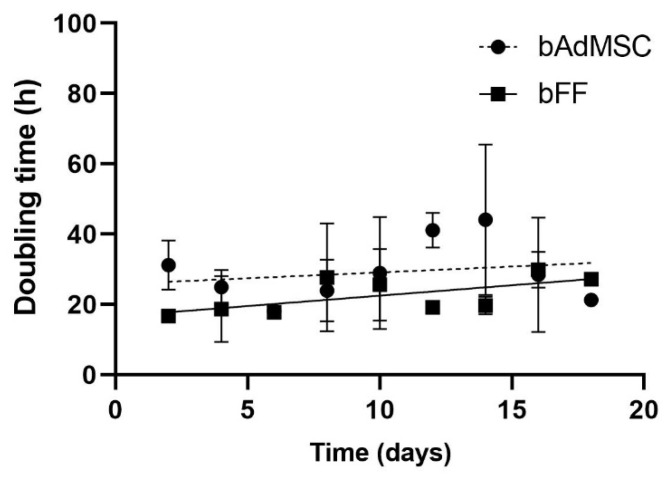
Bovine mesenchymal adipose tissue cells and fetal fibroblasts doubling time analysis using cellular count at every 48 h of culture. bFF cells showed shorter cell doubling compared to bAdMSCs (*p* = 0.0096).

bFFs presented a shorter doubling time (Figure 3), approximately 22 h, compared to adipose tissue cells, which presented a doubling time of approximately 29 h (*p* = 0.0096).

### 3.3. Reprogramming Efficiency and Morphological Analysis of iPSCs

**Figure 4:**
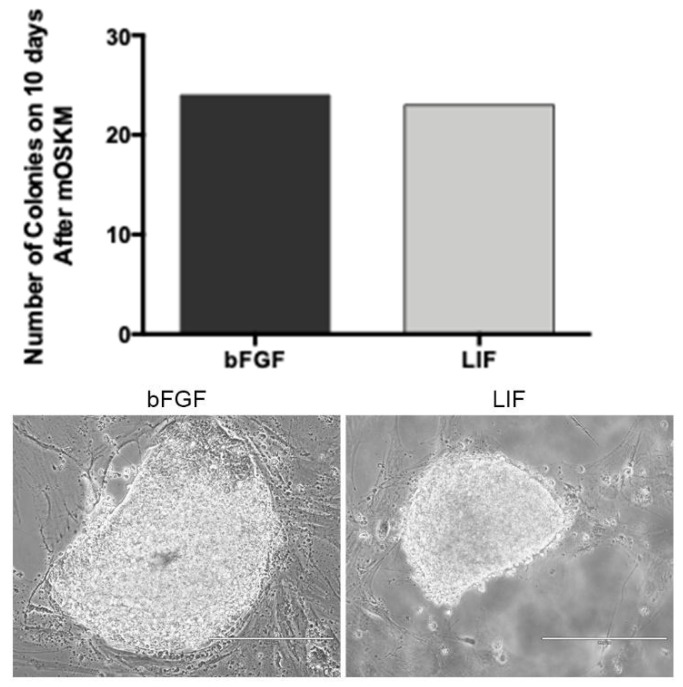
biPSC colonies in different supplementations: bFGF or LIF. The colonies showed a rounded shape and the formation of colonies with delimited borders (Scale bar: 400 μm and 200 μm, respectively).

After pluripotency induction, only bFF cells showed morphological signs of reprogramming 10 days after transduction. The reprogrammed cells were cultured in different supplements, bFGF or LIF, and were analyzed regarding their morphology (Figure 4). The reprogramming process supplemented with bFGF showed 24 colonies (22 AP positive), and those supplemented with LIF presented 23 colonies (21 AP positive, Table 1). In both conditions of supplementation, the cells presented a round shape and the formation of colonies with defined borders.

**Figure 5:**
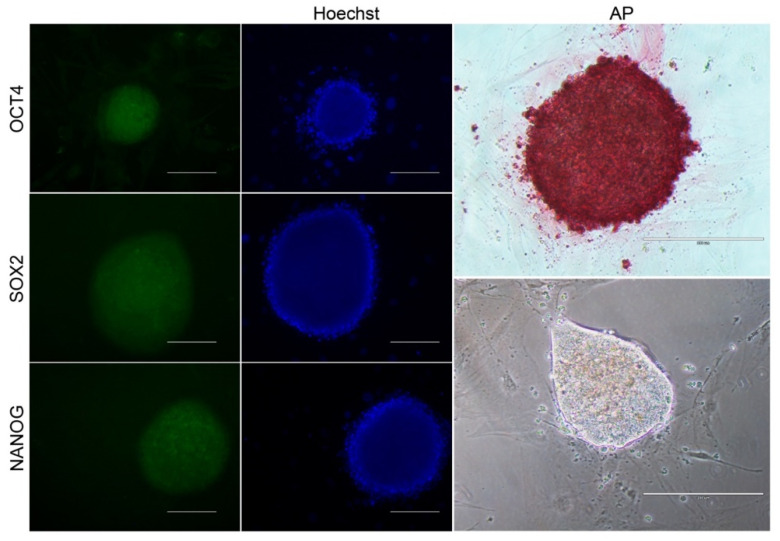
Immunocytochemistry for AP, OCT4, SOX2, and NANOG in biPSCs supplemented with LIF (Scale bar 200 μm).

Colonies supplemented with LIF showed compacted colonies with well-defined borders and, based on their morphology, were selected for further characterization, such as the detection of alkaline phosphatase (AP) and markers related to the pluripotency: NANOG, OCT4, and SOX2 (Figure 5).

## 4. Discussion

Bovine iPSCs have already been reported in several divergent studies regarding reprogramming methods and culture conditions, and the majority were derived from fetal or embryonic bovine fibroblasts [18,36]. In the present study, we characterized fetal bovine fibroblasts and mesenchymal cells derived from adipose tissue and reprogrammed these cells for the generation of biPSCs.

The bFFs and bAdMSCs were positive for CD90, which is typical of mesenchymal stem cells [37], and the cells were negative for pluripotency surface markers (SSEA-1, SSEA-3, and SSEA-4). As seen in previous work on adipose tissue cells and fibroblasts of different animal species, cells can differentiate into mesodermal lineages [15]. Similar to these results, the bFFs and bAdMSCs were able to differentiate into chondrocytes.

Faster cell proliferation rates are among the main characteristics of stem cells [38]. In this study, bFFs presented a shorter doubling time, which means a higher proliferation rate, showing that these cells need approximately 22 h to double their population. In comparison, the bAdMSCs needed approximately 29 h. Since the cells were kept at the same passage, such difference may be associated with the origin of the cell or even the donor’s age, as seen in other species [15,18,39,40], and might affect the reprogramming efficiency of the cell lines analyzed [41].

Interestingly, after inducing the bFFs and bAdMSCs into pluripotency, only biPSCs derived from bFF cells were generated. Thus, besides the reprogramming process being multifactorial dependent, to our knowledge, there are no reports of iPSCs derived from bAdMSCs. Ruiz et al. [41] reported that the higher proliferation rate of the origin cell is a necessary event required for the acquisition and maintenance of pluripotency in the human model, similar to our results.

After pluripotency induction, the bFF cells showed clear signs of cell reprogramming, such as dome-shaped and compact colonies with well-defined borders, similar to those previously reported by Bessi and collaborators [42] with bFGF or bFGF + LIF +2i supplementation. In addition, we detected the pluripotency markers OCT4, SOX2, NANOG, and alkaline phosphatase, and further analyses are still desirable to confirm the true pluripotent status of these cells.

To maintain the pluripotency of biPSCs during the in vitro culture, supplementation is necessary. LIF is responsible for inhibiting differentiation and stimulating self-renewal [43], while bFGF regulates self-renewal and differentiation [44]. Several authors have already reported the dependence of bFGF + LIF for the maintenance of biPSCs in vitro [20,28,30]; however, other reports have reported the culture of biPSCs bFGF + LIF + 2i (MEKi: PD0325901 and GSK3i: CHIR99021) [42] or only bFGF [18,26]. The biPSCs generated in this study were maintained for at least 21 passages in culture supplemented with bFGF or LIF. In addition, no morphological differences during the reprogramming process were observed between the different medium supplementations in this study. Whereas the exact mechanisms behind the different media requirements necessary to maintain the pluripotency of iPSCs generated from different cell lines have not yet been elucidated in large animal models, we have experienced similar outcomes in previous studies of other species [12,15,18].

Our study demonstrated that the efficiency in generating biPSCs cells might be influenced by the origin of the somatic cells and possibly the donor age. The biPSCs generated from bFF cells were characterized based on their morphology and immunophenotype. These bovine reprogrammed cells can be used in translational studies to improve animal reproduction and for veterinary or human regenerative medicine.

In summary, our results showed that the bFFs and bAdMSCs have the potential to differentiate into chondrocytes after the in vitro induction, were CD90 positive, and the bFFs presented a shorter doubling time. The bAdMSCs, however, showed a higher doubling time rate and no signs of reprogramming. In our conditions, the bFFs seem more promising for in vitro cellular reprogramming, as they presented expected morphological changes such as the formation of well-defined colonies, and the putative iPSCs generated were positive for alkaline phosphatase and markers related to pluripotency (OCT4, SOX2, and NANOG). Nonetheless, additional morphological analysis including more animals, and molecular analyses of the data presented here, could further characterize the state of the pluripotency of generated cells and complement the results gathered here. Finally, we conclude that multiple aspects, such as the cell tissue source, the age of the animal, and the proliferation rates can influence the generation of biPSCs. Moreover, our results can contribute to the development of pluripotent cells in large animal models.

## Figures and Tables

**Table 1:** Number of colonies (total and AP positive) observed 10 days after the transduction protocol.

	Total Colonies	AP Positive Colonies	AP Positive/Total %
bFGF	24	22	91.66%
LIF	23	21	91.30%

## Data Availability

Not applicable.
